# Repeat Blood Culture Positive for *B. pseudomallei* Indicates an Increased Risk of Death from Melioidosis

**DOI:** 10.4269/ajtmh.2011.10-0618

**Published:** 2011-06-01

**Authors:** Direk Limmathurotsakul, Vanaporn Wuthiekanun, Gumphol Wongsuvan, Sukanya Pangmee, Premjit Amornchai, Prapit Teparrakkul, Nittaya Teerawattanasook, Nicholas P. J. Day, Sharon J. Peacock

**Affiliations:** Department of Tropical Hygiene, Department of Microbiology and Immunology and Mahidol-Oxford Tropical Medicine Research Unit, Faculty of Tropical Medicine, Mahidol University, Bangkok, Thailand; Department of Medicine and Department of Clinical Pathology, Sappasithiprasong Hospital, Ubon Ratchathani, Thailand; Centre for Clinical Vaccinology and Tropical Medicine, Nuffield Department of Clinical Medicine, University of Oxford, Churchill Hospital, Oxford, United Kingdom; Department of Medicine, Cambridge University, Addenbrooke's Hospital, Cambridge, United Kingdom

## Abstract

Melioidosis, a bacterial infection caused by *Burkholderia pseudomallei*, is notoriously difficult to cure despite appropriate antimicrobial therapy and has a mortality rate of up to 40%. We demonstrate that a blood culture positive for *B. pseudomallei* taken at the end of the first and/or second week after hospitalization for melioidosis is a strong prognostic factor for death (adjusted odds ratio = 4.2, 95% confidence interval = 2.1–8.7, *P* < 0.001 and adjusted odds ratio = 2.6, 95% confidence interval = 1.1–6.0, *P* = 0.03, respectively). However, repeat cultures of respiratory secretions, urine, throat swabs, or pus/surface swabs provide no prognostic information. This finding highlights the need for follow-up blood cultures in patients with melioidosis.

Melioidosis is a community-acquired infectious disease caused by the Gram-negative bacillus *Burkholderia pseudomallei*.^1^ This organism is present in the environment in areas where melioidosis is endemic, and infection is acquired through skin inoculation, inhalation or ingestion. Melioidosis is the most common cause of community-acquired bacteremic pneumonia in northern Australia^2^ and the third most frequent cause of death from infection diseases in northeastern Thailand (after human immunodeficiency virus/acquired immunodeficiency syndrome and tuberculosis).^1^ Common manifestations include pneumonia, hepatosplenic abscesses, septic arthritis, parotid and prostatic abscesses, and urinary tract infection. Persons with suspected melioidosis require hospital admission cultures of blood, respiratory secretions, urine, throat swab, pus if abscesses are identified and accessible, and swabs from any skin lesions. *Burkholderia pseudomallei* is not part of the normal human flora, and isolation of this organism from any specimen is diagnostic for melioidosis.

Death occurs in 40% of patients with melioidosis in our setting.^1^ Several factors have been identified that are associated with a higher risk of death, including older age, pneumonia, markers of organ dysfunction, positive blood culture, and high quantitative count of *B. pseudomallei* in blood or urine.^3^,^4^ Follow-up cultures are easy to perform and relatively inexpensive, but there is limited information on the significance of positive *B. pseudomallei* cultures over time and whether these provide additional prognostic information. We evaluated the hypothesis that follow-up qualitative and/or quantitative culture can be used to predict survival outcome in patients with melioidosis.

The patients described here were consecutive individuals presenting to Sappasithiprasong Hospital between January 1, 1997 and December 31, 2006 with culture-confirmed melioidosis who were identified as part of on-going surveillance associated with clinical trials. The hospital diagnostic laboratory was contacted daily to identify patients with one or more cultures positive for *B. pseudomallei*. Rounds of the medical and intensive care wards were conducted daily to identify additional cases. Any patient not already known to us who we suspected of having melioidosis on the basis of admission clinical features had samples obtained for culture, including blood, respiratory secretions (sputum, or tracheal aspirate if intubated), urine, throat swab, pus, and surface swabs from skin lesions.

Microbiologic specimens were processed as described previously.^5^ All patients with microbiologically confirmed melioidosis were subsequently visited daily to obtain information on history, examination findings, and outcome (defined as survival to discharge or in-hospital death). Repeat blood culture was performed at the end of the first and second weeks after diagnosis in patients who were failing to improve despite parenteral antimicrobial therapy. In addition, other specimen types that were positive for *B. pseudomallei* at admission were obtained every week until negative results were obtained, and patients in whom new signs and symptoms developed and were suspected of new organ involvement had a repeat screening of relevant clinical specimens.

For purposes of analysis, clinical specimens obtained within the first three days were grouped as admission samples, those obtained during days 4–10 as first follow-up samples, and those obtained after day 10 as second follow-up samples. A few relatives took moribund patients home to die and these patients were assumed to have died.

Ethical approval for all clinical trials was obtained from the Ethical and Scientific Review Subcommittee of Thai Ministry of Public Health.^6^–^9^ Quantitative culture of blood, urine, sputum, and pus was performed in adult patients (> 18 years of age) during 2004–2006 as described.^10^

Simple and multivariable logistic regression analysis was used to examine the association between culture result and outcome. Sex, age, history of diabetes, pneumonia, hypotension, and duration of in-hospital intravenous antimicrobial treatment received were included in the multivariable analysis. Drugs used were amoxicillin/clavulanate, ceftazidime, and carbapenems. Fisher's exact test and the Mann-Whitney test were used to compare categorical variables and continuous variable between groups, respectively.

There were 2,243 patients admitted to Sappasithiprasong Hospital during 1997–2006 with their first episode of culture-confirmed melioidosis. Of these patients, 1,314 (59%) were male and 929 (41%) were female. Median age was 49 years (interquartile range [IQR] = 37–60 years), and 212 patients (9%) were less than 15 years of age. Death occurred in 956 patients (43%).

On admission, blood, respiratory secretions, urine, throat swab, and pus/surface swab were obtained for culture from 1,997, 841, 1,152, 874, and 862 patients, respectively (Table 1). Of these patients, blood, respiratory secretions, urine, throat swab, and pus/surface swab culture were positive for *B. pseudomallei* in 1,160 (58%), 550 (65%), 255 (22%), 324 (37%) and 761 (85%) patients, respectively. Having a blood, respiratory secretion, urine, or throat swab culture obtained on admission that was positive for *B. pseudomallei* was strongly associated with death (*P* ≤ 0.01, for all specimens) (Table 1). Having a pus/surface swab culture obtained on admission that was positive for *B. pseudomallei* was associated with a survival outcome, and most of these patients (58%, 443 of 761 patients) had only one localized lesion. Associations between death and a *B. pseudomallei*-positive respiratory secretion, urine, or throat swab culture on admission was independent of bacteremia (Table 2).

Follow-up blood cultures were performed during days 4–10 in 321 patients and after day 10 in 190 patients. Patients who had a blood culture positive for *B. pseudomallei* on admission were more likely to have a positive follow-up blood culture than patients who had a negative admission blood culture (31% [50 of 159] versus 10% [16 of 162]; *P* < 0.001). The median duration of antimicrobial drugs received in patients who had a positive follow-up blood culture was not different from that of patients who had a negative follow-up blood culture (7 days [IQR = 5–7 days] versus 7 days [IQR = 4–7 days]; *P* > 0.1). Multivariable logistic regression demonstrated that follow-up blood cultures positive for *B. pseudomallei* were strongly associated with death (adjusted odds ratio = 4.2, 95% confidence interval = 2.1–8.7, *P* < 0.001 and adjusted odds ratio = 2.6, 95% confidence interval = 1.1–6.0, *P* = 0.03, respectively). However, this association was not observed in follow-up culture of other specimen types (Table 1).

The prognostic value of follow-up blood culture was evaluated further in a subset of patients who had quantitative blood culture. Thirteen patients had quantitative blood culture on admission and one or more follow-up cultures (Figure 1). All 13 patients received ceftazidime or a carbapenem drug within the first two days after admission, and a decrease in bacterial count in blood was observed in all patients. Most patients with a positive quantitative follow-up blood culture (80%, 4 of 5 patients) had an admission bacterial count in blood greater than 1 colony-forming unit (CFU)/mL, and most patients with a negative quantitative follow-up blood culture (88%, 7 of 8 patients) had an admission bacterial count in blood of less than 1 CFU/mL (*P* = 0.03). Of these 13 patients, two patients who had a bacterial count in blood ≤ 100 CFU/mL at admission (patients A and B), and one patient who had persistent bacteremia (0.2 CFU/mL on day 2, 0.1 CFU/mL on day 17, and 0.1 CFU/mL on day 26; patient D) died (Figure 1). Patient D was a 66-year old man with diabetes mellitus and non-Hodgkin lymphoma, and was post-chemotherapy. The patient who had a follow-up blood culture of 4.7 CFU/mL on day 9 (patient C) improved and was transferred to continue parenteral therapy at a community hospital.

**Figure 1.**
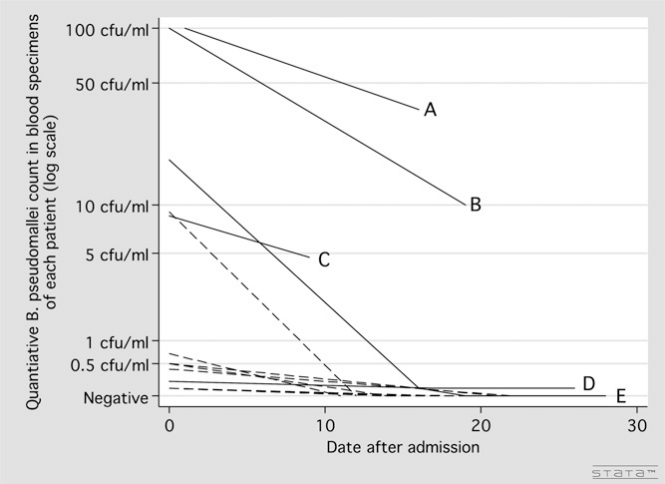
Quantitative *Burkholderia pseudomallei* count in blood specimens obtained from 13 patients who had follow-up quantitative hemoculture performed. Each line represents the results of one patient. Solid lines represent five patients who had follow-up hemoculture positive for *B. pseudomallei* (patients A–E), and broken lines represent eight patients who had follow-up hemoculture negative for *B. pseudomallei*. Of 13 patients, three patients (A, B, and D) died. Patient C improved and was transferred to continue parenteral antimicrobial therapy at a community hospital. Patient E had a positive follow-up blood culture of 0.1 colony-forming units/mL on day 16, but negative blood cultures on days 19 and 28.

Our study shows that follow-up blood culture is an important predictor of late in-patient death from melioidosis. This finding agrees with observations for other infectious diseases.^11^,^12^ Response to antimicrobial therapy for melioidosis is often slow (mean duration of fever of 9 days),^13^ and markers for patients who survive the first week of in-patient care but remain at risk of death are not well established. Our study indicates that patients with melioidosis should receive weekly follow-up blood culture until clearance of the organism from blood

Our results from a small subset of patients who had follow-up quantitative blood culture indicated that positive follow-up blood culture was associated with several factors, including high bacterial load on admission and an immunocompromise condition associated with a presumed failure to eradicate the infecting bacterium. We found that most of the patients who had follow-up positive blood culture had a bacterial count in blood of more than 1 CFU/mL on admission. However, one patient who had a bacterial count in blood of 0.2 CFU/mL failed to clear the organism from the blood (patient D). This finding could have been caused by his markedly impaired immune function and defective phagocytic cell function associated with chemotherapy. This finding emphasizes that in the clinical setting, intensive and detailed management is required of patients with positive follow-up blood culture, even without data on quantitative count.

Although a positive culture of respiratory secretions, urine, or sputum on admission was associated with death, this association was not observed for follow-up cultures of these specimen types. Positive cultures of respiratory secretions and urine on admission represent organ involvement, including pneumonia and urinary tract infection, which have been shown to be independent prognostic factors for death.^3^

This study extends previous findings by describing the value of a throat swab on admission as a prognostic factor, an observation that was independent of blood and urine culture results (as demonstrated by multivariable logistic regression). Furthermore, a throat swab is better than a sputum culture because it can be performed in every patient and not only patients with respiratory symptoms and sputum production. Lack of an association between death and persistently positive respiratory secretions, urine, throat swab, and pus/surface swab is new evidence supporting previous observations.^14^ This finding adds to our experience that a small number of patients with persistent culture of these specimens can nonetheless be cured with oral eradicative treatment.^14^ Localized lesions are difficult to eradicate and slow to resolve, but routine follow-up culture of the related sample types does not appear to have any clinical utility.

A limitation of this study is that not all clinical specimens were obtained from every patient. For example, there were only 779 patients with respiratory symptoms who had respiratory secretions and blood obtained for culture at admission (Table 2). In addition, follow-up cultures were not performed in every patient, and the timing of repeat cultures was variable. Nonetheless, these limitations do not affect the main conclusion of this study that blood culture should be followed up at least weekly in melioidosis patients and until a negative result is observed.

## Figures and Tables

**Table 1:** Prognostic value of a positive culture of blood, respiratory secretion, urine, throat swab, and pus/surface swab culture on admission, first follow-up sampling period (4–10 days after admission), and second follow-up sampling period (after day 10) in patients with melioidosis, Thailand*

Sample type and timing	No. patients who died/total no. patients, (% mortality rate)	Crude OR (95% CI)	Adjusted OR† (95% CI)	*P*‡
Blood				
At hospitalization				
Positive	733/1,160 (63.2)	7.0 (5.7–8.7)	5.1 (4.0–6.6)	< 0.001
Negative	164/837 (19.6)			
During first follow-up sampling period				
Positive	25/66 (37.9)	3.6 (2.0–6.6)	4.2 (2.1–8.7)	< 0.001
Negative	37/255 (14.5)			
During second follow-up sampling period				
Positive	18/37 (48.7)	3.1 (1.5–6.5)	2.6 (1.1–6.0)	0.03
Negative	36/153 (23.5)			
Respiratory secretion				
At hospitalization				
Positive	284/550 (51.6)	1.8 (1.4–2.5)	1.8 (1.1–2.8)	0.01
Negative	107/291 (36.8)			
During first follow-up sampling period				
Positive	11/69 (15.9)	0.6 (0.2–1.5)	0.4 (0.1–1.5)	> 0.1
Negative	9/36 (25.0)			
During second follow-up sampling period				
Positive	9/35 (25.7)	1.7 (0.5–5.3)	4.9 (0.8–31)	0.09
Negative	6/35 (17.1)			
Urine				
At hospitalization				
Positive	147/255 (57.7)	2.9 (2.2–3.9)	2.1 (1.5–3.0)	< 0.001
Negative	284/897 (31.7)			
During first follow-up sampling period				
Positive	4/16 (25.0)	1.0 (0.3–3.5)	1.9 (0.4–8.3)	> 0.1
Negative	26/107 (24.3)			
During second follow-up sampling period				
Positive	1/6 (16.7)	0.6 (0.1–5.5)	0.3 (0.0–5.8)	> 0.1
Negative	12/47 (25.5)			
Throat swab				
At hospitalization				
Positive	127/324 (39.2)	3.7 (2.7–5.2)	3.1 (2.0–4.8)	< 0.001
Negative	81/550 (14.7)			
During first follow-up sampling period				
Positive	3/23 (13.0)	1.1 (0.2–4.9)	1.1 (0.2–7.1)	> 0.1
Negative	5/40 (12.5)			
During second follow-up sampling period				
Positive	3/11 (27.3)	7.1 (1.0–49.8)	21 (0.5–871)	> 0.1
Negative	2/40 (5.0)			
Pus/surface swab				
At hospitalization				
Positive	130/761 (17.1)	0.5 (0.3–0.8)	0.5 (0.3–0.9)	0.01
Negative	37/131 (28.2)			
During first follow-up sampling period				
Positive	18/114 (15.8)	2.1 (0.7–6.5)	1.5 (0.4–5.5)	> 0.1
Negative	4/48 (8.3)			
During second follow-up sampling period				
Positive	12/92 (13.0)	4.8 (0.6–38.5)	6.7 (0.7–60)	0.09
Negative	1/33 (3.0)			

* OR = odds ratio; CI = confidence interval; Positive = positive for *Burkholderia pseudomallei*; Negative = negative for *B. pseudomallei.*

† OR was adjusted for blood culture positive for *B*. *pseudomallei*, sex, age, history of diabetes, pneumonia, hypotension, and duration of in-hospital intravenous antimicrobial treatment received.

‡ *P* value for adjusted OR.

**Table 2:** Prognostic significance of a respiratory secretion, urine, throat swab, and pus/surface swab culture positive for *Burkholderia pseudomallei* on admission, adjusted for blood culture positive for B. pseudomallei*

Sample type	Patients with positive blood culture (%)	Patients with negative blood culture (%)	Adjusted OR† (95% CI)	*P*‡
Positive respiratory secretions	224/386 (58.0)	276/393 (70.2)	2.3 (1.4–3.7)	0.001
Positive urine	152/564 (27.0)	82/545 (15.1)	1.9 (1.3–2.7)	0.001
Positive throat swab	149/361 (41.3)	171/497 (34.4)	3.1 (2.0–4.9)	< 0.001
Positive pus/surface swab	205/257 (79.8)	392/462 (84.9)	0.7 (0.4–1.2)	> 0.1

* OR = odds ratio; CI = confidence interval; Positive = positive for *B*. *pseudomallei*; Negative = negative for *B*. *pseudomallei.*

† OR was adjusted for blood culture positive for *B*. *pseudomallei*, sex, age, history of diabetes, pneumonia, hypotension, and duration of in-hospital intravenous antimicrobial treatment received.

‡ *P* value for adjusted OR.
